# Early life group 2 innate lymphoid cells in health and disease

**DOI:** 10.1186/s40348-025-00209-w

**Published:** 2026-01-03

**Authors:** Claudia U. Duerr, Marcus A. Mall

**Affiliations:** 1 Department of Microbiology, Infectious Diseases and Immunology, Charité - Universitätsmedizin Berlin, corporate member of Freie Universität Berlin and Humboldt-Universität, Berlin, Germany; 2 Department of Pediatric Respiratory Medicine, Immunology and Critical Care Medicine, Charité - Universitätsmedizin Berlin, corporate member of Freie Universität Berlin and Humboldt-Universität, Berlin, Germany; 3 German Center for Child and Adolescent Health (DZKJ), Partner Site Berlin, Berlin, Germany; 4 German Center for Lung Research (DZL), Associated Partner Site Berlin, Berlin, Germany; 5 Cluster of Excellence ImmunoPreCept, Charité - Universitätsmedizin Berlin, Berlin, Germany

**Keywords:** Innate immunity, Barrier surfaces, Early life, ILC2, Human, Health, Disease

## Abstract

ILC2s are innate lymphoid cells that become activated by alarmins and are major producers of type 2 signature cytokines. In mice and human, ILC2s have been identified and characterized in several pre-clinical disease models and patients with a spectrum of diseases. Interest in the regulation and function of ILC2s has grown substantially in recent years due to their capability to act as first responders to external and internal stimuli and their contribution to tissue immunity in health and disease. Importantly, ILC2s are present early on during ontogeny, long lived and can orchestrate immune and non-immune cell populations highlighting their potential impact early and late in life. However, the impact of ILC2s is only starting to be appreciated in early life immune responses. Here, we provide an overview of ILC2s in childhood and adolescence in health and disease. We further discuss the (potential) modulation of early life ILC2s and their clinical implications for therapeutic treatments.

## Introduction

Group 2 innate lymphoid cells (ILC2s) are regarded as the innate equivalent of T helper 2 cells (Th2 cells) and orchestrate tissue immunity and thereby contribute to immune defense and maintaining homeostasis. ILC2s are present in different tissues including the lung, intestine and skin, but are also found in blood. Circulating ILC2s are discussed as biomarkers in the context of inflammatory diseases. Due to their characteristics of being present early on in ontogeny and their longevity, ILC2s are increasingly appreciated in early life immunology.

ILC2s are a subgroup of ILCs. ILCs belong, similar to T lymphocytes, to the lymphoid lineage sharing lineage-defining transcription factors and effector cytokines. However, ILCs are missing an antigen specific receptor for activation. In contrast, ILC are activated in an antigen-independent manner which is mainly driven by chemokines, cytokines and other modulators of immune responses. ILCs are classified into two subsets: NK cells representing the innate counterpart to cytotoxic CD8 T cells and helper ILCs. Helper ILCs are Group1 (ILC1), Group2 (ILC2) and Group 3 (ILC3) and serve as the innate equivalent to Th1, Th2 and Th17 cells [1]. ILC1s are characterized by the expression of the transcription factor Tbet and secretion of IFN-$\gamma$, the signature transcription factor of ILC2s is GATA3 and the effector cytokines are IL-4, IL-5 and IL-13. ILC3s depend on ROR$\gamma$t to produce IL-17 and IL-22 [2]. ILC2s have been described in detail in 2010 in several studies investigating mouse models at steady state and of type 2 inflammation [3–5], but earlier evidence of a type 2 response triggered by a cell population of non-T and non-B cells has been reported in mice and human [6, 7]. ILC2s are also currently discussed as biomarker for diagnostics and therapeutic purposes.

It is becoming more and more evident that early life immune status and immunological events impact health later in life. Importantly, in both, mice [8] and humans [9], ILC2s are present already at the pre- and neonatal period, long lived and as such regarded as first sensors, amplifiers and signaling hubs shaping innate and adaptive tissue immunity. As such ILC2s are increasingly appreciated in early life basic and clinical research. We will summarize current literature of ILC2s in childhood and adolescence providing an overview for life science researchers and clinicians on this fascinating immune cell population linked to tissue immunity, resilience and health.

### Type 2 immune responses and ILC2 functions – detrimental or beneficial?

Type 2 immunity is characterized by the presence of signature cytokines IL-4, IL-5, IL-9 and IL-13 and can act on immune as well as non-immune cells. Type 2 responses involve many different cell types; key sources of Type 2 signature cytokines and thus important modulators are innate ILC2s as well as adaptive Th2 cells. Protective in parasitic infections by triggering the “weep and sweep” response, but detrimental in the globally rising incidence of allergic inflammatory diseases, modulation of type 2 immunity is regarded as a key interest in basic as well as in clinical research. Besides its role in parasitic infections and allergies, type 2 immunity was further explored firstly by the LIAR (Local Immunity And/or Remodeling/Repair in both health and disease) hypothesis [10] focusing on the function of eosinophils and by several reports expanding the view of type 2 immunity to additional advantageous responses such as the immune response against insect venoms [11] or to behavior in an allergen avoidance specific manner [12, 13]. However, these studies were restricted to adaptive antigen-specific Th 2 cells. In contrast to Th2 cells, ILC2s are activated by modulators of immune responses such as alarmins and are as such direct sensors of their microenvironment, first responders and amplifiers of signals. The potential influence of early life ILC2s on behavior, whether in the context of allergen avoidance or general social behaviors, was observed in adult mice [14]. The importance of early life ILC2s for tissue homeostasis in humans is just starting to be investigated. Higher levels of early life ILC2s are present in the intestine of children triggering regenerative processes and tissue growth [15]. Whether this observation applies to pulmonary ILC2s in early life, particularly in specific clinical settings or developmental stages, remains unclear. Further research is needed to clarify whether ILC2s play a protective or harmful role in pediatric conditions.

### Identification of ILC2s in humans

Shortly after the detailed reports of mouse ILC2s [3–5], the description of human ILC2s followed [16]. Interestingly, in these first reports ILC2s were investigated in fetal gut and lung tissue [16]. ILC2s were detected by their typical characteristics: the response to cytokines and other modulators of immune responses of their environment since ILC2s do lack an antigen specific receptor. Moreover, there are many overlaps in surface markers with adaptive T lymphocytes. Similar to T cells, ILC2s express the respective receptor chains and are therefore able not only to respond to cytokines including IL-2 and IL-7, but also to prostaglandin D2. Prostaglandin D2 is detected by CRTH2 (**C**hemoattractant **R**eceptor homologous molecule expressed on **TH2** cells), which represents a key characteristics of human ILC2s [16]. Of note, characteristic ILC2 markers can be modulated by immune status and tissue location and thus exhibit slightly different expression profiles due to tissue imprinting on ILC2 phenotype [17].

### Early life ILC2s at steady state

ILC2s are present in newborn blood with a higher presence in boys compared to girls. Interestingly, a heterogenous expression of c-kit, a receptor for stem-cell factor linked to hematopoiesis, was detected on those early life ILC2s [18] similar to blood ILC2s in adults [19, 20]. Notably, variations in c-kit expression are associated with alterations in ILC2 biology. In addition, human ILC2s are detectable in several fetal tissues such as lung and gut [16] and are enriched in the circulation in children compared to adults [21, 22]. In the infant intestine specifically, ILC2s exhibit an increased proliferative state already at steady state. This characteristic is thought to be needed for the increased tissue regeneration at the time indicating that enhanced proliferation is not always linked to pathology but to an early life phenotype [15]. In mice, the ontogeny of ILC2s is better understood than in humans with low numbers of ILC2s being present before birth and an increase of ILC2s in different tissues and waves during life [23]. Mouse ILC2s are also able to migrate during adult life from peripheral organs and the bone marrow upon challenge through both the blood and lymphatic systems [24–26]. Interestingly, the ILC2 activating alarmin IL-33 correlates with Type 2 signature cytokines in the blood of preterm infants [27]. Blood but also tissue, contains ILC progenitors that are already present at steady state [20, 28]. Of note, the increase of ILC2s in lung tissue is triggered by the first breath which induces the alarmin IL-33 [29]. Further, an increase in activity status such as proliferation and shaping dendritic cell activation in early life was reported [30, 31]. Pulmonary but also intestinal tissue exhibit an increase of ILC2s in the early life of mice similar to humans [32]. This enhanced dynamics and stronger activation of mouse ILC2s in early life at mucosal barriers has recently been extended to data in human infant intestine [15].

### Early life ILC2s upon viral infection

ILC2s in children and young adults have been studied in several different inflammatory and infectious diseases. Children born with human immunodeficiency virus (HIV) show an accelerated disease progression compared to adults. Antiretroviral therapy decreases morbidity and mortality, viremia and immune cell dysfunction related to HIV infection in children. Adaptive immunity in pediatric patients is limited to control virus spread pointing towards a functional importance of ILCs in this setting. However, pediatric patients infected with HIV have reduced circulating ILC2s in the blood [22, 33]. The reason for this drop in ILC2s is unclear since ILC2s are not known to express receptors needed for HIV entry. Indirect mechanisms such as ILC2 inhibition by viral induced interferon [34] could account for this phenotype. Only when long-term antiretroviral therapy is started at birth, ILCs are restored contrary to T helper cells [22]. In contrast to adults, affected children exhibit not only decreased number of ILCs in the circulation and tonsils but also changes in metabolic and activation status [22] highlighting how early changes in immunological status can affect innate immune cell populations during life.

Respiratory viral infections trigger mainly a type 1 immune response, however, ILC2s are induced upon respiratory viral infections such as influenza virus (IV) by IL-33 [35]. IL-33 is expressed by both non-immune and immune cells. During helminth infection, non-hematopoietic epithelial cell-derived IL-33, triggers ILC2 activation, while myeloid-derived IL-33 is critical for the regulation of adaptive T cells [36]. It is currently unclear whether a comparable mechanism occurs in IAV infection. In children, infections with respiratory syncytial virus (RSV) are very common. Higher levels of ILC2s have been reported in nasal aspirates of children with severe RSV bronchiolitis [37]. Of note, ILC2s are reduced upon viral infection such as by SARS-CoV2 in adult but also pediatric patients [21, 38]. Virus induced mechanisms, namely interferon, can act on ILC2s to restrain their activity [34, 39, 40]. The response to interferon is dampened not only in adult asthmatic patients but also in children [41]. Interestingly an increased interferon response during SARS-CoV2 infection has been reported in children [42, 43], however, whether ILC2s from pediatric patients respond in the same or similar manner to interferon still needs to be investigated.

### Early life ILC2s in atopic and other chronic inflammatory diseases

Although ILC2s are present and modulated in the blood in chronic inflammatory diseases, mucosal barriers are regarded as their predominant niche. Indeed, nasal polyps of chronic rhinosinusitis patients were investigated in early ILC2 studies [44]. A recent report aimed to analyze ILC2s in nasal scraping of children suffering from chronic rhinosinusitis. ILC2s were increased in atopic children, however, additional studies with larger sample sizes are needed for more conclusive analyses [45]. Upon allergen exposure of adult patients suffering from allergic rhinitis (hay fever), increased levels of CRTH2^+^ ILC2s were reported in the circulation [46]. Increased percentages of ILC2s were also found already in children with allergic rhinitis [47].

In the lungs, ILC2s have been implicated in the pathogenesis of type 2 airway inflammation in patients with asthma. Interestingly, in asthma patients, sputum but also circulatory CRTH2^+^ ILC2s are present at higher numbers and exhibit increased activity [48–50]. Also in children, specifically when suffering from severe therapy-resistant asthma (STRA), not only ILC2s in general are increased in blood and sputum, but also IL-13 expressing ILC2s [51, 52]. Upon successful therapeutic treatment with steroids, the levels of ILC2s are decreased indicating a direct link between the level of ILC2s and disease severity. Indeed, ILC2s have been discussed for some time as biomarkers for adult patients with type 2 airway inflammation [53].

Interestingly, ILC2s within inflamed tissue of chronic obstructive pulmonary disease (COPD) and idiopathic pulmonary fibrosis (IPF) patients are found to exhibit slightly different phenotypic characteristics: here ILC2s have been reported to be either positive for CRTH2 or ST2 (IL-33 receptor chain). Reasons for that could be imprinting by their microenvironment [54], regulation of receptors triggering ILC2 migration [55] or cross-regulation of other unknown factors modulated in an inflamed pulmonary microenvironment.

**Fig. 1:**
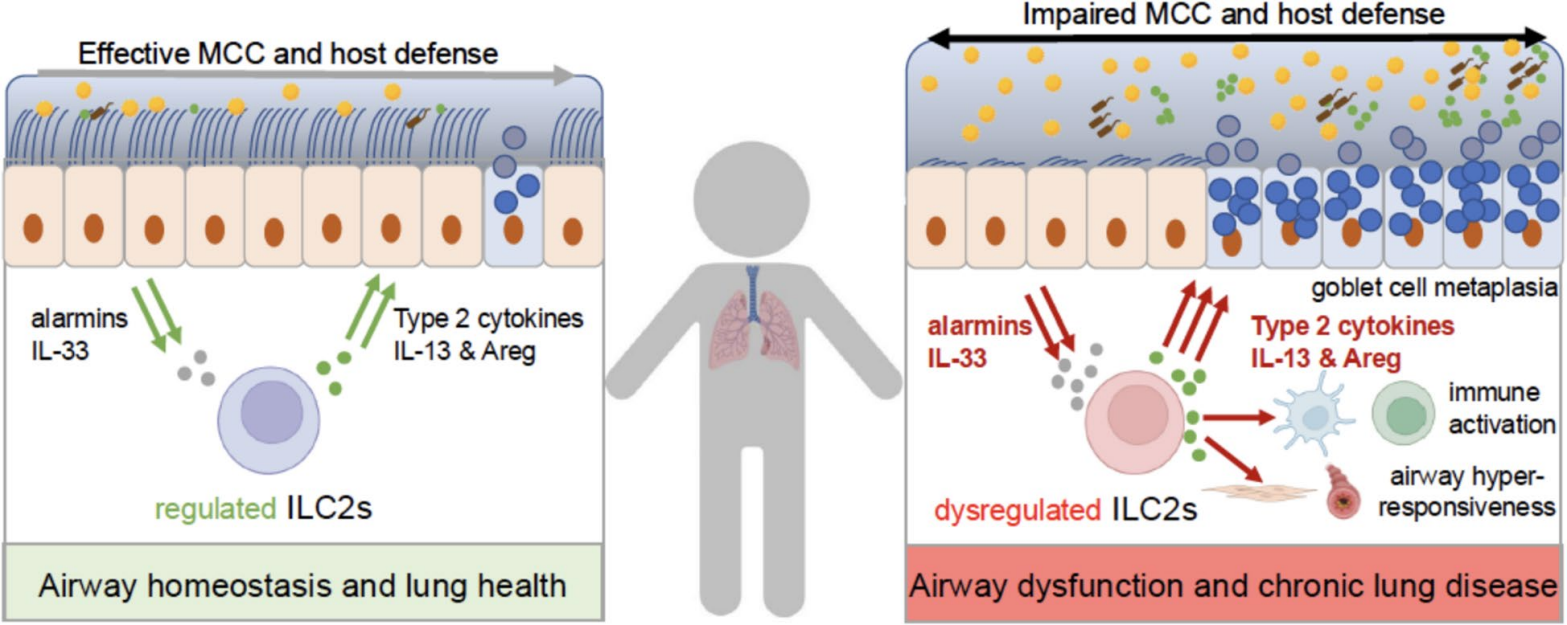
Tissue ILC2s regulate airway homeostasis and lung health. Tissue resident ILC2s are active at steady state, expressing signature cytokines and contributing to airway homeostasis and lung health. However, upon dysregulation, ILC2s act on epithelial cells, triggering goblet cell metaplasia and mucus hypersecretion, on immune cells including dendritic cells and T-cells leading to chronic airway inflammation, and on smooth muscle cells leading to airway hyperresponsiveness. Collectively, these effects cause airway dysfunction and chronic lung disease. Figure created by the authors with selected elements from BioRender.com

The rare genetic disease cystic fibrosis (CF) is caused by mutations in the gene encoding the cystic fibrosis transmembrane conductance regulator (CFTR) functioning as a cAMP-regulated epithelial anion channel responsible for transepithelial secretion of chloride, bicarbonate and fluid that is essential for proper mucus function and effective mucociliary clearance of inhaled pathogens or irritants from the airways, thus playing an important role in mucosal defense and homeostasis of the lungs [56]. In patients with CF, CFTR dysfunction leads to impaired mucociliary dysfunction and a severe lung disease characterized by airway mucus plugging, chronic airway infection and inflammation that causes progressive structural lung damage and ultimately death due to respiratory failure [56, 57]. Although CF lung disease starts in the first months of life [58] studies of ILC2s remain limited to adult CF patients with advanced lung disease, where reduced levels of circulatory ILC2s were reported and discussed to be a consequence of an increased tissue homing of active ILC2s to the lungs [59]. However, studies in mice with airway-specific overexpression of the $\beta$-subunit of the epithelial sodium channel ($\beta$ENaC) that phenocopy CF-like airway mucus dehydration and impaired mucociliary clearance and develop key features of CF lung disease from the first weeks of life suggest [60–62] that ILC2s may be implicated in the orchestration of early airway inflammation in children with CF [60, 63].Interestingly, these studies showed that impaired mucociliary clearance triggers spontaneous type 2 inflammation that is characterized by an increase of ILC2s that is further aggravated by allergen challenge in the lungs of young $\beta$ENaC-transgenic mice compared to their wild-type littermates [61, 63]. In these studies, it was also shown that these ILC2s secrete IL-13, thereby contributing to airflow obstruction and inflammation via multiple mechanisms including its well-known roles in airway smooth muscle contraction, goblet cell metaplasia and mucus hypersecretion predicted to aggravate intraluminal mucus plugging [64, 65] and alternative activation of lung resident macrophages, which play an important role in early inflammation and lung damage in this model of CF lung disease [63, 66–68]. Collectively, the data from this murine model suggest that ILC2s, probably via sensing noxious agents that accumulate in the airways when mucociliary clearance is impaired and transmitting signals to surrounding stromal as well as innate and adaptive immune cells, may play an important role in the complex in vivo pathogenesis of airway inflammation CF lung disease in early life (Fig. 1). However, future studies including children with CF will be needed to test this hypothesis. In addition, it will be interesting to determine the role of ILC2s in other muco-obstructive lung diseases associated with impaired mucociliary clearance such as primary ciliary dyskinesia (PCD) and non-CF bronchiectasis [69, 70].

ILC2s have been detected in the skin of healthy individuals but also adults suffering from atopic dermatitis, [71–73]. Interestingly, treatment with the IL-4R binding antibody dupilumab reduces the number of ILC2s in high responders compared to low responders [74]. Since successful treatment of atopic dermatitis with dupilumab has also been reported in children [75], it would be of great interest to see whether ILC2s follow the same pattern in children as in adults.

Inflammatory bowel diseases (IBD) affecting the mucosal barrier of the intestine/gut are on the rise in young adults [76]. Interestingly, an increase in cases is mainly attributed to changes in life style which could be diet dependent. ILC2s can be modulated by dietary changes [77, 78]. An inulin fiber diet triggered IL-5 expressing ILC2s by increased microbiota derived secondary bile acids. As a result, eosinophil levels and intestinal inflammation were increased in inulin fiber diet fed mice. Importantly, IBD patients showed a similar molecular and cellular pattern such as an enhanced level of bile acids and increased eosinophils indicating a role for pathologic ILC2 responses also in humans [78]. However, whether this diet and its impact on the microbiota-ILC2 axis might also be involved in the increased number of young patients with IBD is still unknown.

ILC2 research has been heavily focused on atopic disease but is increasingly extended to non-atopic settings. ILC2s were reported in several cancer models (reviewed in [79]). The knowledge of ILC2s in cancer patients is currently very limited and is mainly based on data from adults [80]. Another understudied area are ILC2s in autoimmune diseases with first reports associating ILC2s with diseases such as lupus nephritis [81]. Interestingly, 20% of patients suffering from systemic lupus erythematosus (SLE) are diagnosed during childhood or adolescence and lupus nephritits is more frequent and severe in pediatric patients [82]. However, whether ILC2s have a role in autoimmune diseases in children and young adults still needs to be investigated.

### Modulation of ILC2s by early life events and consequences for health later in life

It is becoming more and more evident that events and conditions in early life impact immune responses, resilience and thus overall health. As already pointed out above, several atopic and chronic inflammatory diseases are diagnosed in early childhood and it is accepted that sensitization occurs early in life [83]. Variables leading to different hypothesis are currently discussed and experimentally challenged [84]. An important concept is the developmental origins of health and disease (DOHaD) approach. Here, different factors, conditions and events during early life including prenatal and perinatal development shape health and disease later in life. Interestingly, DOHaD is strongly linked to epigenetic changes that are happening as consequences upon exposures or perturbations and imprint the offspring. DOHaD was originally proclaimed by David Barker and colleagues investigating the impact of nutritional access during gestational periods to glucose [85]. Until now, the consequences of access to nutrition during gestation on ILC2s are unknown. However, diet and ILC2 activity has been linked in adult mice [86, 87] and humans [77, 78] emphasizing a possible link of prenatal ILC2 function via modulation by maternal diet.

An interesting aspect of DOHaD is the mismatching theory, which has its origin in evolution biology. Here, characteristics that have been developed in a specific environment are no longer an advantage in a different, changed environment. Applied to DOHaD, the embryo with very limited nutrients will adapt to this situation and imprint this in a lifelong manner. However later in life with access to an abundance of nutrients, the body is not able to deal with the increased intake of food, such as glucose as described by Barker and colleagues [88, 89]. However, until today it is understudied, how much the mismatching theory can be applied to microbial and/or immunological mechanisms.

Pharmacological interventions such as antibiotic treatments during pregnancy are linked to the risk of developing inflammatory diseases such as asthma. Gestational antibiotic treatment was shown to reduce the levels of short chain fatty acids (SCFA) and modulate the interferon responses resulting in increased ILC2 activation in the offspring [90]. Interestingly, increased SCFA were observed in mice fed a high fiber diet resulting in reduced susceptibility to allergic airway inflammation in the offspring [91]. Moreover, supplementation of the maternal diet resulted in changes of the microbial composition in the airways of infants [92]. It is tempting to speculate that ILC2 function may have been affected here as well.

Viral infections during pregnancy can directly affect the health of the newborn and child [93]. In a mouse model, viral infection was mimicked in dams by treatment with the double-stranded RNA analog poly (I:C) resulting in an increased ILC2 activity and type 2 pathology in the offspring [94]. Together, these studies in mouse models highlight that dysregulated prenatal ILC2 function can result in severe health issues in vivo. However, future studies including humans will be necessary to elucidate to what extent maternal diet and exposure to antibiotics and viral infections impact ILC2s activity in children and health later in life.

### Conclusions and outlook

ILC2s are major producers of type 2 cytokines and can be found in the circulation as well as in tissues from infancy onward in health. ILC2s are associated not only with atopic and other chronic inflammatory diseases, but also with viral infections. Most of the current literature studies ILC2 biology in adulthood and we are just beginning to understand some of their contribution and regulatory capacity in early life. Changes in the microbiome early in life can impact health later on. It is therefore tempting to speculate that the function and metabolic state of ILC2s may be influenced by microbiome modulation in the neonatal period and childhood. Recent studies have initiated the discussion on the application of ILC2s as a potential tool for immunotherapy due to their immunomodulatory characteristics. Reports include the expression of IL-10 or even granzyme B by ILC2s under specific conditions [95–98]. Whether human ILC2s in early life may exhibit similar a pattern and potential needs to be explored in future studies. Moreover, in addition to ILC2s, other ILC subsets are present during early life and unravelling the specific functions and relative importance of these different subsets will be important for a comprehensive understanding of the role of ILCs in health and disease.

## Data Availability

Not applicable.

## Abbreviations

CF: Cystic fibrosis
CFTR: Cystic fibrosis transmembrane conductance regulator
COPD: Chronic obstructive pulmonary disease
CRTH2: Chemoattractant receptor homologous molecule expressed on TH2 cells
DOHaD: Developmental origins of health and disease
GZMB: Granzyme B
GVHD: Graft-versus-host disease
HIV: Human immunodeficiency virus
ILC2: Group 2 innate lymphoid cells
IPF: Idiopathic pulmonary fibrosis
IV: Influenza virus
NK cells: Natural killer cells
OXPHOS: Oxidative phosphorylation
PCD: Primary ciliary dyskinesia
RSV: Respiratory syncytial virus
SCFA: Short chain fatty acid
STRA: Severe therapy-resistant asthma
